# Chromosome-Scale Genome and Comparative Transcriptomic Analysis Reveal Transcriptional Regulators of β-Carotene Biosynthesis in Mango

**DOI:** 10.3389/fpls.2021.749108

**Published:** 2021-10-12

**Authors:** Xiaowei Ma, Xiang Luo, Yongzan Wei, Tuanhui Bai, Jiangli Shi, Bing Zheng, Wentian Xu, Li Li, Songbiao Wang, Jisen Zhang, Hongxia Wu

**Affiliations:** ^1^Key Laboratory for Postharvest Physiology and Technology of Tropical Horticultural Products of Hainan Province, South Subtropical Crops Research Institute, Chinese Academy of Tropical Agricultural Sciences, Zhanjiang, China; ^2^State Key Laboratory of Crop Stress Adaption and Improvement, Henan University, Kaifeng, China; ^3^College of Horticulture, Henan Agricultural University, Zhengzhou, China; ^4^Center for Genomics and Biotechnology, Fujian Agriculture and Forestry University, Fuzhou, China

**Keywords:** mango, genome, β-Carotene, transcriptional regulation 2, fruit ripening

## Abstract

Mango (2*n* = 2*x* = 40) is an important tropical/subtropical evergreen fruit tree grown worldwide and yields nutritionally rich and high-value fruits. Here, a high-quality mango genome (396 Mb, contig N50 = 1.03 Mb) was assembled using the cultivar “Irwin” from Florida, USA. A total of 97.19% of the sequences were anchored to 20 chromosomes, including 36,756 protein-coding genes. We compared the β-carotene content, in two different cultivars (“Irwin” and “Baixiangya”) and growth periods. The variation in β-carotene content mainly affected fruit flesh color. Additionally, transcriptome analysis identified genes related to β-carotene biosynthesis. *MiPSY1* was proved to be a key gene regulating β-carotene biosynthesis. Weighted gene co-expression network analysis, dual luciferase, and yeast one-hybrid assays confirmed that transcription factors (TFs) MibZIP66 and MibHLH45 activate *MiPSY1* transcription by directly binding to the CACGTG motif of the *MiPSY1* promoter. However, the two TFs showed no significant synergistic effect on promoter activity. The results of the current study provide a genomic platform for studying the molecular basis of the flesh color of mango fruit.

## Introduction

Mango (*Mangifera indica* L.) fruit is one of the most nutritious and highly-priced edible fruit worldwide. It possesses a diploid genome (2*n* = 2*x* = 40 chromosomes) and belongs to the Sapindales order, Anacardiaceae family, and *Mangifera* genus. Mango is grown on 2.5 million hectares in tropical and subtropical areas of the world, with an annual production of ~46.6 million (Wang et al., 2020). It is the fifth most economically important fruit crop, followed by bananas, grapes, apples, and oranges (Deshpande et al., 2017). Its yellow flesh is rich in carotenoids, an important natural pigment involved in plant growth and adaptation that the immune functions of humans and reduces the occurrence of diseases such as cancer (Sun et al., 2017). Thus, understanding the molecular basis of flesh color is the main goal for the genetic improvement of mangoes. The plastidial 2-C-methyl-d-erythritol 4-phosphate pathway, which is responsible for carotenoid biosynthesis, is highly conserved in plants (Liu et al., 2015). However, the regulatory mechanisms governing the expression of genes of this pathway are complex and vary among plant species. To date, only a few plant transcription factors (TFs), such as phytochrome-interacting factor 1 (PIF1) in *Arabidopsis* (Toledo-Ortiz et al., 2010), SlSGR1 (encoding the protein STAY GREEN) in tomato (Luo et al., 2013), and *CsMADS6* (Mcm1-Agamous-Deficiens-Srf) and *CsMADS5* in citrus (Lu et al., 2018), have been shown to directly regulate the expression of carotenoid biosynthesis genes. However, some TFs, including *SINAC4, TAGLl, ERF6, GLK2*, and *CubHLHl*, affect carotenoid metabolism through other pathways, such as fruit development, ethylene biosynthesis, and photomorphogenesis (Chung et al., 2010; Karlova et al., 2011). It is still unclear whether such TFs directly mediate carotenoid metabolism (Sagawa et al., 2015; Su et al., 2015). Previous studies have shown that although carotenoid composition is relatively stable in different mango cultivars, carotenoid content varies greatly among cultivars (Mercadante and Rodriguez-Amaya, 1998; Ma et al., 2018). β-carotene is the predominant carotenoid that affects the flesh color of mature mango fruit (Ma et al., 2018). Nevertheless, little is known about the transcriptional mechanisms of that regulated β-carotene biosynthesis and hence influence the flesh color of mango fruit.

Owing to the long history of cultivation, wide geographical distribution, and intense selection, there are more than a thousand mango cultivars worldwide. Genetic analysis of mango germplasm diversity has shown that the whole mango group can be divided into two subgroups: Indian and Southeast Asia groups (Warschefsky and Wettberg, 2019). Recently, the genomes of Indian cultivars (variety “Alphonso”) (Wang et al., 2020) and Southeast Asian cultivars (variety “Hong Xiang Ya”) (Li et al., 2020) have been sequenced, expanding our understanding of mango genome evolution. The Florida mango cultivars are generally considered hybrids between Indian cultivars and Southeast Asian cultivars. The Florida variety “Irwin” has excellent production stability and environmental adaptability (Olano et al., 2005). Additionally, “Irwin” mangoes have higher economic value compared to “Alphonso” and “Hong Xiang Ya” owing to their red peel and low fiber-containing flesh. Thus, “Irwin” is considered an elite maternal parent widely used for mango breeding in recent decades and is genetically much more diverse than other cultivars. However, a high-quality reference genome of Florida cultivar mangoes had not been published until now.

In this study, we sequenced and analyzed the genome of the mango cultivar “Irwin” using single-molecule real-time (SMRT) sequencing and high-throughput chromosome conformation capture (Hi-C) techniques. We subsequently performed genome and comprehensive transcriptome analyses of genes involved in carotenoid biosynthesis to explore the molecular mechanism of β-carotene biosynthesis and its regulation during fruit development and ripening. Additionally, we identified important TFs controlling the expression of a key structural gene involved in the β-carotene synthesis. Accordingly, we described a high-quality cultivar genome and elucidated the genetic basis for the flesh color of mango fruit.

## Materials and Methods

### Materials

Two *M. indica* cultivars “Irwin” (IR, deep yellow-fleshed) and “Baixiangya” (BY, pale yellow-fleshed), widely cultivated in China, were chosen from the orchard of the South Subtropical Crops Research Institute, Zhanjiang (110°4′E, 21°12′N), Guangdong Province, China, for this study.

### Genome Sequencing

Genomic DNA was extracted from the fresh young leaves of mango cultivar “Irwin” according to the modified CTAB method, and used for both Illumina and PacBio sequencing. DNA quantity and quality were assessed using a NanoDrop 2000 Spectrophotometer (NanoDrop Technologies, Wilmington, DE, USA) and electrophoresis on a 0.8% agarose gel. For Illumina sequencing, we constructed a short-read (300 bp) library using the NEBNext Ultra DNA Library Prep Kit (New England BioLabs, Ipswich, MA, USA). For PacBio sequencing, 20-kb SMRTbell libraries were prepared according to the protocol released by PacBio, and then the library was sequenced on the PacBio Sequel System (Biomarker Technologies Corp., Beijing, China) with one SMRT cell, yielding more than 20 Gb of raw data.

### K-Mer Analysis and Genome Assembly

A total of 18.87 Gb high-quality-filtered data were used to perform a 17-kmer analysis. The genome size, heterozygous rate, and repeat rate were estimated using the program Jellyfish (Marçais and Kingsford, 2011). MECAT (version 1.2) (Xiao et al., 2017) was used for the correction and assembly of PacBio data, and Polish (version 1.22) (Walker et al., 2014) was used for the assembly correction with Illumina short reads. To check the completeness of the assembly, the RNA-seq data from fruits were mapped to the genome assembly using BLASTN (version 2.6.0+) (Altschul et al., 1990); various sequence homology and coverage parameters were determined. Furthermore, we evaluated genome completeness using the Benchmarking Universal Single-Copy Orthologs (BUSCO) analysis (Simão et al., 2015). The Hi-C sequencing of the chromosome-level genome assembly was performed according to the previously described method (Belton et al., 2012).

### Genome Annotation

The present genome was annotated with three characteristics. (i) We first used *de novo* prediction and homology-based alignment to predict repetitive sequences across the mango genome. The repeat sequence database was constructed using the LTR_FINDER (Xu and Wang, 2007), PILER-DF (Edgar and Myers, 2005), and RepeatScout (Price et al., 2005). RepeatMasker and RepeatProteinMask software (Tamura, 1992) were used to identify repeat sequences. (ii) We then annotated the predicted protein-coding genes included in the databases, namely, National Center for Biotechnology Information non-redundant database, Gene Ontology, Kyoto Encyclopedia of Genes and Genomes, SwissProt, and EuKaryotic Orthologous Groups. (iii) Finally, we predicted miRNA and snRNA sequences using the INFERNAL software (Nawrocki and Eddy, 2013). The rRNA was identified using the BLAST program, and tRNAscan-SE was used to predict tRNA (Chan and Lower, 2019).

### RNA Sequencing

Healthy fruits of two cultivars (“Irwin” and “Baixiangya”) in three distinct developmental stages were sampled. The developmental stages were: S1, early development stage, 40 days after full boom (DAFB); S2, harvest stage, 86 DAFB; and S3, fully ripe stage, 96 DAFB. The flesh of the collected fruit was separated from the peel and frozen in liquid nitrogen for β-carotene measurement and RNA sequencing. All experiments were performed in triplicate.

Total RNA was extracted from the frozen flesh tissue of each sampling point and each cultivar using a Quick RNA Isolation Kit (Invitrogen, Carlsbad, CA, USA). The cDNA libraries were assembled according to the protocol of the manufacturer of the NEBNext Ultra RNA Library Prep Kit for Illumina (E7530) and NEBNext Multiplex Oligos for Illumina (E7500), and the libraries were sequenced on the Illumina HiSeq XTen platform (Biomarker Technologies Corp., Beijing, China). The DESeq2 software (Robinson et al., 2010) was employed to identify differentially expressed genes, which were filtered according to *p* ≤ 0.05, and expression value >0.

### Analysis of β-Carotene Content

The β-carotene in the flesh was extracted as described by Ma et al. (2018). The carotenoids were analyzed using the Waters Ultra-high-performance liquid chromatography (Milford, MA, USA) with a photodiode array detector and a single quadrupole mass spectrometer detector in series (6120 Quadrupole, Agilent, Santa Clara, CA, USA). The column used was a YMC carotenoid (C30) column (Wilmington, NC, USA). The eluent phases were as follows: mobile phase A, acetonitrile:methanol = 3:1 (v/v); and phase B, 100% methyl tert-butyl ether (MTBE). Each eluent contained 0.01% butylated hydroxytoluene (BHT). Gradient elution was performed as follows: 0–2 min, 85:15 A:B; 2–4 min, 75:25 A:B; 4–7 min, 40:60 A:B; 7–10 min, 40:60 A:B; 10–13 min, 5:95 A:B; 13–23 min, 85:15 A:B. The flow rate was 0.8 ml/min, and the injection volume was 5 μl.

### RNA Extraction and Quantitative Reverse Transcription PCR Analysis

Total RNA was extracted from frozen flesh using TRIzol reagent (TaKaRa, Dalian, China) according to the instructions of the manufacturer. The primers used for real-time PCR were designed using Primer 5 and are listed in Supplementary Table 10. The PCR mixture (20 μl total volume) comprised 10 μl of LightCycler 480 SYBR Green I Master Mix (Roche, Mannheim, Germany), 2 μl of each primer, 2 μl of diluted cDNA, and 6 μl DEPC-H_2_O. Reverse transcription (RT)-PCR) conditions were as follows: pre-incubation at 95°C for 5 min, followed by 45 cycles at 95°C for 5 s, 58°C for 15 s, and 72°C for 10 s. The expression levels of genes of interest were calculated according to the formula 2^−ΔΔCt^.

### Yeast One-Hybrid Assay

Yeast one-hybrid (Y1H) assay was performed according to the Gold Yeast One-Hybrid Library Screening System User Manual (Clontech, Palo Alto, CA, USA). Either the *MiPSY1* or *MiPSY1*-mut promoter was inserted into the Phis2 vector to generate the baits. *MiPSY1*-mut is a mutated version of *MiPSY1* in which the core sequence (CACGTG) of two E-box elements has been altered and is “TCTAGC.” The full-length coding sequences (CDS) of *MibZIP66* and *MibHLH45* were cloned into the pGADT7 vector to generate the prey and were transformed into the yeast strain Y187 containing the bait. The TF–promoter interaction was determined according to the growth ability of the co-transformed yeast cells on synthetic defined -Trp-Leu-His media with 3-amino-1,2,4-triazole.

### Dual-Luciferase Transient Expression Assay

The full-length CDS of *MibZIP66* and *MibHLH45* was cloned into the vector pGreenII0029 62-SK as effectors. Meanwhile, the *MiPSY1* promoter was recombined with pGreenII0800-LUC to create the reporter vector. All vectors were transformed into *Agrobacterium tumefaciens* (GV3101), and the cultures were adjusted to an optical density at OD 600 nm of 0.1 with an infiltration medium containing 100 μM acetosyringone, 0.5 M MES, and 10 mM MgCl_2_. *Agrobacterium* with TFs or promoters was injected into *Nicotiana benthamiana* leaves using a 5-ml injection syringe. Transgenic plants were grown in a glasshouse condition for 3 days. Firefly luciferase (LUC) and Renilla luciferase (REN) were determined using the Dual-Luciferase Reporter Assay System (Promega Corp., Madison, WI, USA). Transcriptional activation was calculated as the ratio of LUC to REN.

### Subcellular Localization Analysis

The full-length coding sequences of *MibZIP66* and *MibHLH45* without the stop codon were amplified using PCR and cloned into the GFP vector, under the control of the CaMV 35S promoter. The vector constructs of 35S-MibZIP66-GFP and 35S-MibHLH45-GFP were transformed into *Agrobacterium tumefaciens* strain GV3101 through electroporation and then injected into tobacco leaves. *MiPSY1* or the control vector (pYBA1132) plasmid was co-transformed with a plasmid coding for a mitochondrial marker red fluorescent protein (RFP) into *Arabidopsis* protoplasts by polyethylene glycol (PEG) transformation method according to Lister et al. (2007). Localizations of *MiPSY1, MibZIP66*, and *MibHLH45* were examined using a fluorescence microscope (Zeiss Axioskop 2 Plus; Oberkochen, Germany).

## Results

### Genome Sequencing and Assembly

**Figure 1:**
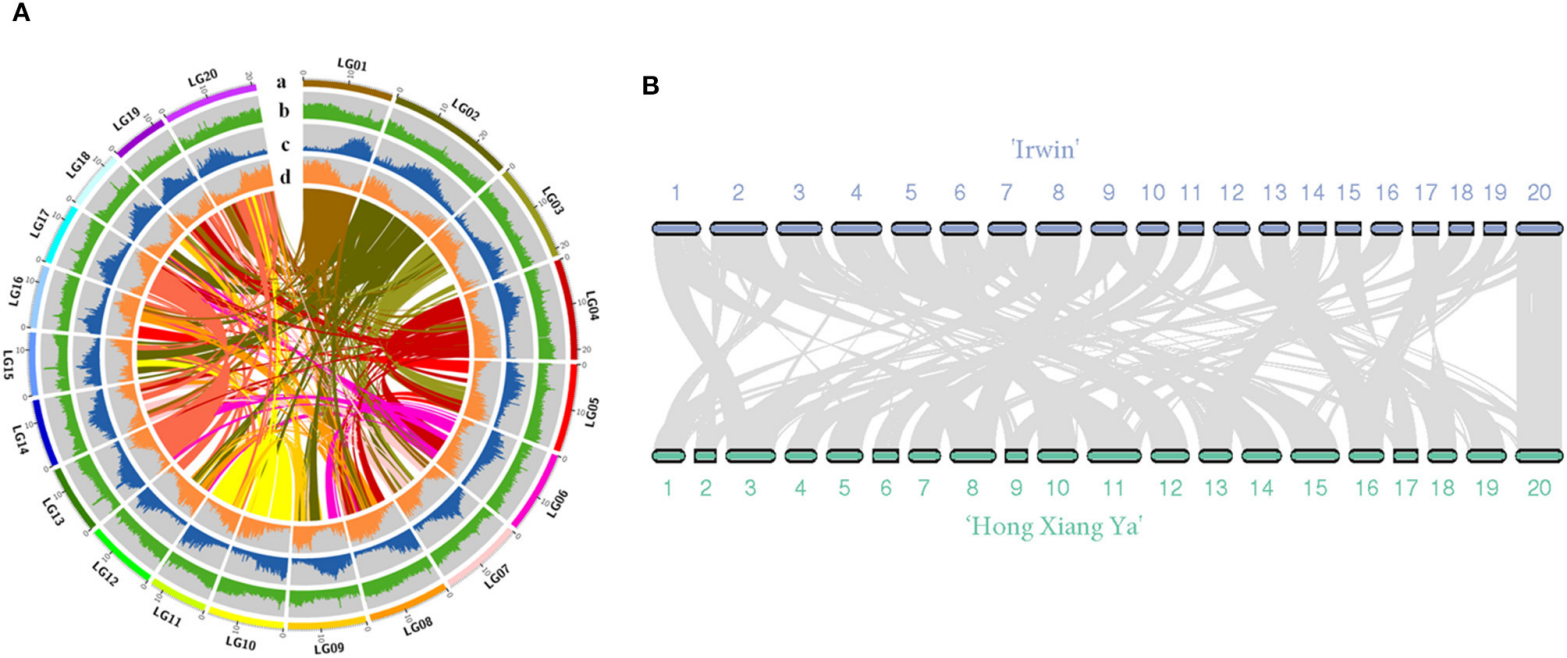
**(A)** Overview of the “Irwin” genome. (a) pseudomolecules, (b) GC content (per 200 kb), (c) repeat density (per 200 kb), (d) gene density; **(B)** Synteny view of the “Irwin” and “Hong Xiang Ya” genomes.

The genome of the mango cultivar “Irwin” was initially estimated to be 327 Mb with a 1.37% heterozygosity rate and 47.28% of the repeat sequence ratio based on the 17-mer sequence (Supplementary Figure 1 and Supplementary Table 2). In total, 20.26 Gb of sequencing data were produced with an average coverage depth of 61.96× using the PacBio single molecular long-read method (Supplementary Table 1). After heterozygosis was accounted for, a draft genome of 396 Mb was assembled, with N50 of 1.03 Mb (Table 1). The assembly of our genome was comparable to that of the recently published genome of the traditional Indian cultivar “Alphonso” (392.9 Mb) (Wang et al., 2020), but better than that of the Southeast Asian variety “Hong Xiang Ya” (~372 Mb) (Li et al., 2020). Then, Hi-C contact frequency derived from Hi-C sequencing was used to order and orient the polished contigs into Hi-C scaffolds. As a result, a total of 97.19% (375 Mb) sequences in the mango genome were anchored to 20 chromosomes, and the chromosome size ranged from 12.8 to 29.8 Mb (Supplementary Tables 3, 4, and Figure 1A). These results are consistent with those of studies by Li et al. (2020) and Wang et al. (2020). Two independent methods were used to evaluate assembly quality. The alignment rates of the RNA-seq reads from the three fruit developmental stages of the two cultivars were ~90.29 and 95.87% (Supplementary Table 5). In contrast, the BUSCO assay showed that ~94% of the core eukaryotic genes (1,991) were retrieved in the assembly, further confirming the continuity and high quality of the assembled genome (Supplementary Table 6). Furthermore, we performed the whole-genome alignment of our assembly and previous mango assembly. One-to-one syntenic blocks showed ~79.61% of our genome sequence matched 84.75% of the “Hong Xiang Ya” genome sequence (Figure 1B), indicating our genome sequence was substantially more complete.

**Table 1:** Statistics of mango genome assembly.

	**With heterozygosis**	**Without heterozygosis**
Number of contigs	3,022	1,305
Length of contigs (bp)	471,122,396	396,192,158
Minimum length of contig (bp)	11,830	12,183
Maximum length of contig (bp)	13,145,087	13,145,087
Average length of contig (bp)	155,897	303,595
N50 (bp)	788,704	1,034,750

### Repetitive Elements and Gene Annotation

We identified a total of 138.29 Mb of repeated sequences, representing 34.92% of the assembled mango genome. Among these repetitive elements, 12.77% were unknown repetitive sequences. The most abundant repeat type was long terminal repeats (LTRs), accounting for 15.49% of the genome. Additionally, we also found long interspersed nuclear elements (LINEs, 0.57%) and DNA transposons (6.25%) (Supplementary Table 7). Combined *de novo* prediction, protein-based homology searches, and RNA-seq analysis identified a total of 36,756 protein-coding genes with an average coding sequence length of 1,142 bp and 5.5 exons per gene (Supplementary Table 8). Moreover, a total of 109 rRNAs, 75 snRNAs, 483 tRNAs, 279 snoRNA, and 131 miRNAs were identified.

### Expression of Carotenoid Genes During Fruit Development and Ripening

**Figure 2:**
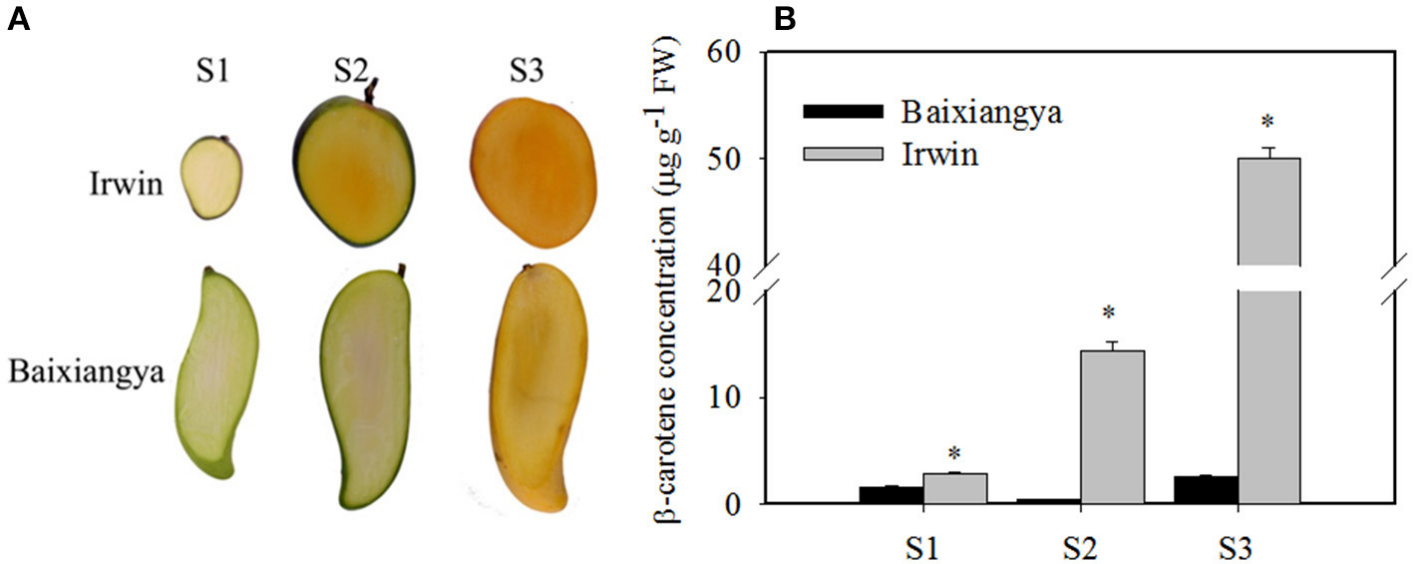
Changes in the color **(A)** and β-carotene concentration **(B)** in the flesh of “Baixiangya” and “Irwin” during fruit development and ripening. Fruit development and ripening were divided into three stages: S1, early development stage; S2, harvest stage; and S3, fully ripe stage. A black asterisk indicates a significant change (*p* ≤ 0.01) between two cultivars.

Studying the changes in β-carotene and the regulation of its biosynthesis will provide insight into the genetic basis of mango fruit flesh color. During fruit development and ripening, β-carotene content increased in “Irwin” mangoes, but remained low in “Baixiangya” mangoes. At the fully ripe stage, β-carotene concentrations in “Irwin” flesh was up to 50.06 μg/g of fresh weight, but was only 2.64 μg of fresh weight in “Baixiangya” flesh (Figure 2B). This difference in β-carotene concentration was also evident visually by the distinct color of the fruits (Figure 2A).

**Figure 3:**
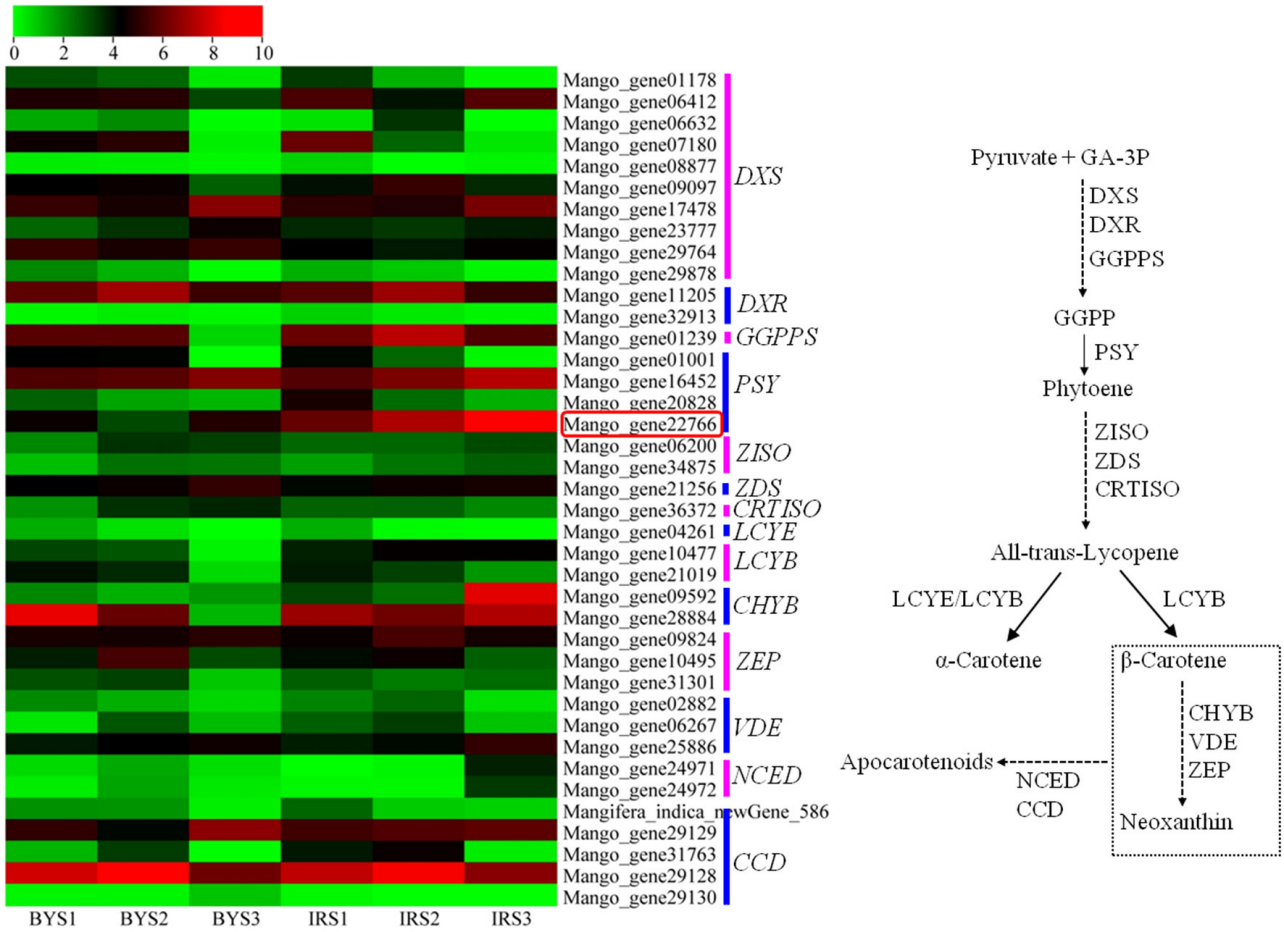
Heatmap of the expression levels of genes related to carotenoid biosynthesis in the flesh of “Baixiangya” (BY) and “Irwin” (IR) during fruit development and ripening. Gene expression was indicated by reads per kilobase per million mapped reads (RPKM) values. Scaled log2 transcript values are shown from green to red, indicating low to high expression, respectively. A schematic of the carotenoid biosynthetic pathway in plants. DXS, 1-deoxy-d-xylulose-5-phosphate synthase; DXR, 1-deoxy-d-xylulose-5-phosphate reductoisomerase; *GGPPS*, geranylgeranyl diphosphate synthase; PSY, phytoene synthase; PDS, phytoene desaturase; ZISO, ζ-carotene isomerase; ZDS, ζ-carotene desaturase; CRTISO, carotene isomerase; LCYE, lycopene ε-cyclase; LCYB, lycopene β-cyclase; CHYB, β-carotene hydroxylase; ZEP, zeaxanthin epoxidase; VDE, violaxanthin de-epoxidase; NCED, 9-*cis*-epoxycarotenoiddioxygenase; CCD, carotenoid cleavage dioxygenase.

**Figure 4:**
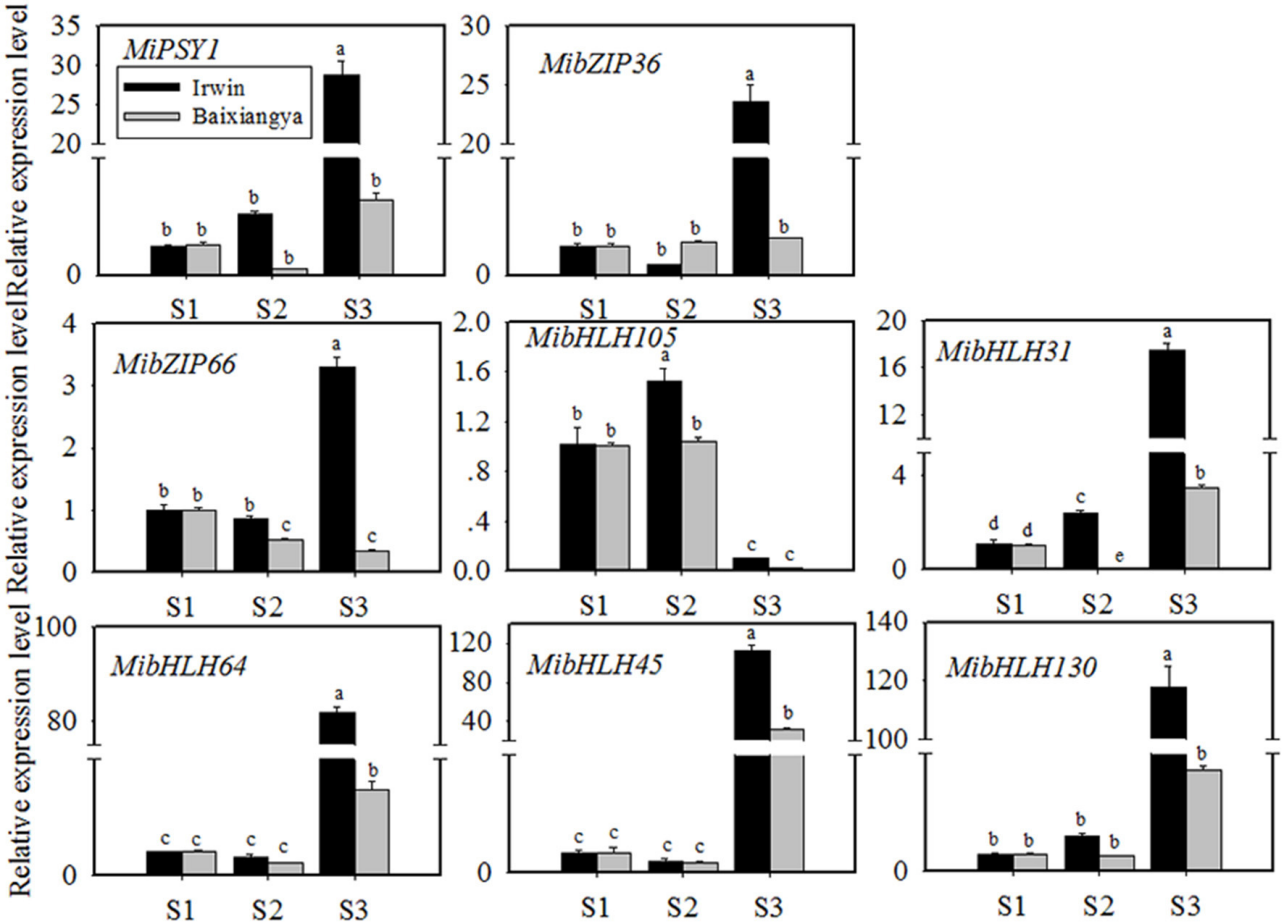
Relative expression levels of *MiPSY1* and seven selected transcription factors (TFs) in the flesh of “Baixiangya” and “Irwin” mangoes during fruit development and ripening. Gene expression levels were analyzed by quantitative reverse transcription analysis. Different letters show significant differences in expression level as calculated by Duncan statistical analysis (*p* < 0.01).

Comparative RNA-seq analysis was performed between “Irwin” and “Baixiangya” mangoes to study the expression profiles of β-carotene biosynthesis-related genes. A total of 52 genes were found to be associated with the carotenoid biosynthesis pathway, and 39 genes were differentially expressed between three developmental stages in both cultivars (Figure 3). Among these differentially expressed genes, the expression levels of *MiDXS* (*Mango_gene17478* and *Mango_gene23777*, encoding 1-deoxy-d-xylulose-5-phosphatesynthase) and *MiPSY* (*Mango_gene16452* and *Mango_gene22766*) were relatively low during the early fruit development stage but increased greatly with continued fruit ripening in both cultivars. *LCYB* (*Mango_gene10477*) showed a considerably higher expression level in “Irwin” than in “Baixiangya” mangoes at the early stage of fruit development. During the fruit ripening period, its expression level remained nearly unchanged in “Irwin,” but decreased in “Baixiangya” mangoes. Similarly, *Mango_gene22766* on chromosome 7 showed a markedly high level of expression in “Irwin” during fruit development and ripening but a very low level in “Baixiangya” mangoes. At the fully ripened stage (S3), the *Mango_gene22766* expression level was 14-fold higher in “Irwin” than in “Baixiangya” based on qRT-PCR data (Figure 4). Hence, the high expression level of these genes is likely to lead to the accumulation of β-carotene in the Mango fruit. In contrast, the transcript levels of *MiCCD* (*Mango_gene31763, Mango_gene29128*, and *Mango_indica_newGene_586*) exhibited a significant decline during the fruit maturation phase, indicating a likely negative role in the development of Mango fruit flesh color. According to the annotation of the genome, *Mango_gene22766* plays an important role in driving the metabolic flux toward the β-carotene formation. Therefore, it can be inferred that *Mango_gene22766* may affect color formation by regulating β-carotene production in the fruit flesh. We sequenced two coding sequences of *Mango_gene22766* from the flesh of “Irwin” and “Baixiangya,” and found they were both 1,299 bp long and encoded a peptide of 432 amino acids long (Supplementary Tables 11, 12). Except for the 400th and 417th amino acid, the rest of deduced amino acids were identical between the two cultivars (Supplementary Figure 2).

### Isolation and Transactivation of Candidate TFs on *MiPSY1*

**Figure 5:**
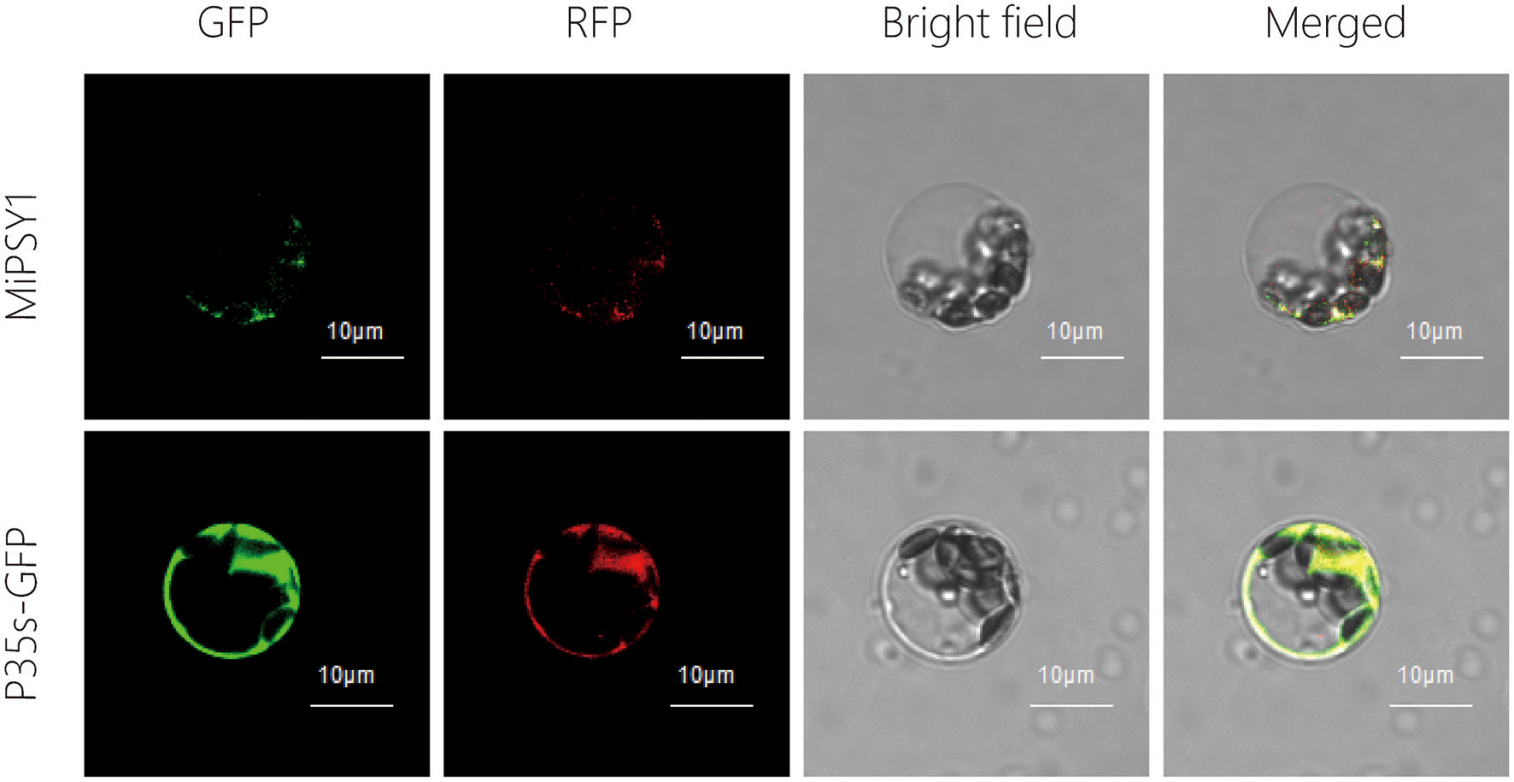
Subcellular location of MiPSY1 protein in *Arabidopsis* protoplasts. Red fluorescent protein (RFP) was included in each transfection to serve as mitochondria localization. Bars = 10 μm.

We named the cloned full-length sequence of Mango_gene22766 as *MiPSY1* (Supplementary Table 11). MiPSY1 was suggested to be distributed in the mitochondria by the WoLF PSORT Prediction online software (http://www.genscript.com/wolf-psort.html). To examine its subcellular location, MiPSY1 was fused to RFP and transiently expressed in *Arabidopsis* protoplasts. Fluorescence microscopy analysis showed that the MiPSY1 protein was localized in the mitochondria (Figure 5). In summary, *MiPSY1* might be a critical candidate gene controlling β-carotene formation in the mango fruit flesh.

To further investigate the potential TFs regulating *MiPSY1* expression, a weighted gene co-expression network analysis of differentially expressed genes was performed (Supplementary Figure 3A). A total of 14 modules were identified (Supplementary Figure 3B). Analysis of module–trait relationships showed that the “MElightcyan” module was significantly correlated with β-carotene content (*r* = 0.86, *p* = 0.00) (Supplementary Figure 3B). Moreover, 49 TFs (including bHLH, bZIP, and NAC) were gathered in this module and were predicted to potentially interact with *MiPSY1* (Supplementary Table 9). According to PLACE and Plant-CARE databases, seven TFs, namely MibZIP36 (Mango_gene12953), MibZIP66 (Mango_gene35447), MibHLH105 (Mango_gene17414), MibHLH31 (Mango_gene17464), MibHLH64 (Mango_gene20675), MibHLH45 (Mango_gene30145), and MibHLH130 (Mango_gene30794), were predicted to be regulatory *cis*-acting elements of the *MiPSY1* promoter (Supplementary Table 13). Thus, we cloned these seven TFs and analyzed their sequences. Further phylogenetic analysis indicated that the seven identified TFs were grouped with *Arabidopsis thaliana* bZIP or bHLH proteins. To confirm the RNA-seq results, the seven TFs identified were further analyzed using qRT-PCR, which validated the RNA-seq results. The transcript levels of the seven TFs were significantly correlated with *MiPSY1* expression and β-carotene content during fruit development and ripening (Figure 4). In summary, these results suggest that these seven TFs might share the regulatory function of *A. thaliana* bZIP or bHLH and act as candidate regulators of β-carotene biosynthesis.

**Figure 6:**
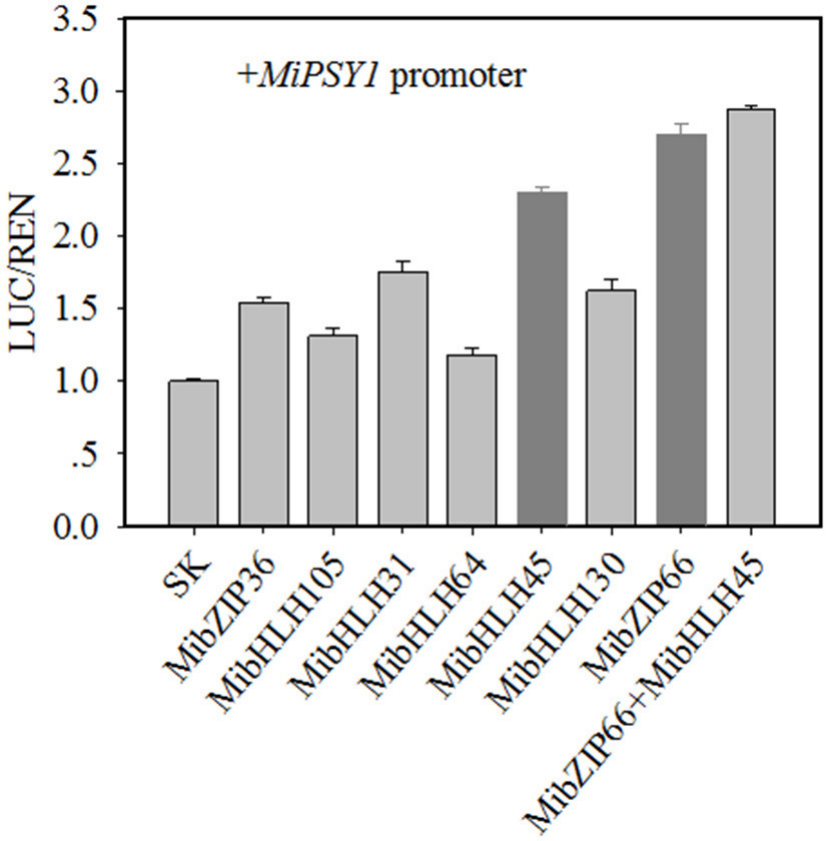
Regulatory effects of selected transcription factors (TFs) on MiPSY1 promoter by dual-luciferase assays. The ratio of luciferase (LUC)/Renilla luciferase (REN) of the empty vector plus promoter was set as 1.

**Figure 7:**
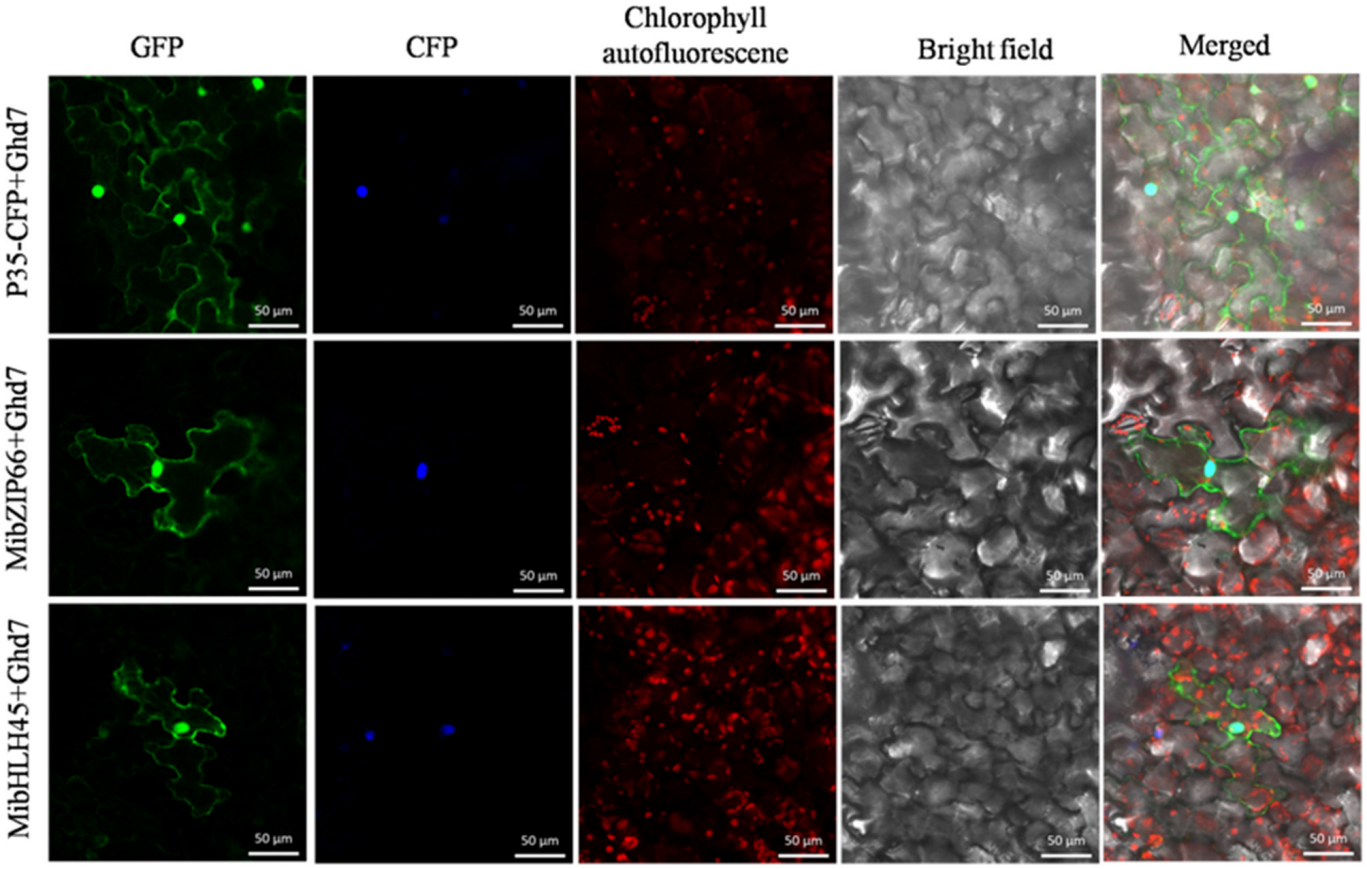
Subcellular location of MibZIP66 and MibHLH45 protein in tobacco (*N. benthamiana*) leaf epidermal cells. The nuclear localization signal-CFP-transformed tobacco epidermis cells served as nuclear markers. The green fluorescence protein was examined at 24 h after inoculation. Bars = 50 μm.

Dual-luciferase transient expression assays were performed to test the potential regulatory effects of the key candidate TFs on the *MiPSY1* promoter. The LUC/REN assays indicated that MibZIP66 and MibHLH45 could significantly activate the *MiPSY1* promoter, with transcription activation increments of 2.7- and 2.3-fold, respectively. The effect of the other five TFs on the *MiPSY1* promoter was limited. The action of MibZIP66 and MibHLH45 in combination had no significant additive effect on the *MiPSY1* promoter activity (Figure 6). MibZIP66 and MibHLH45 were localized exclusively in the nucleus (Figure 7).

**Figure 8:**
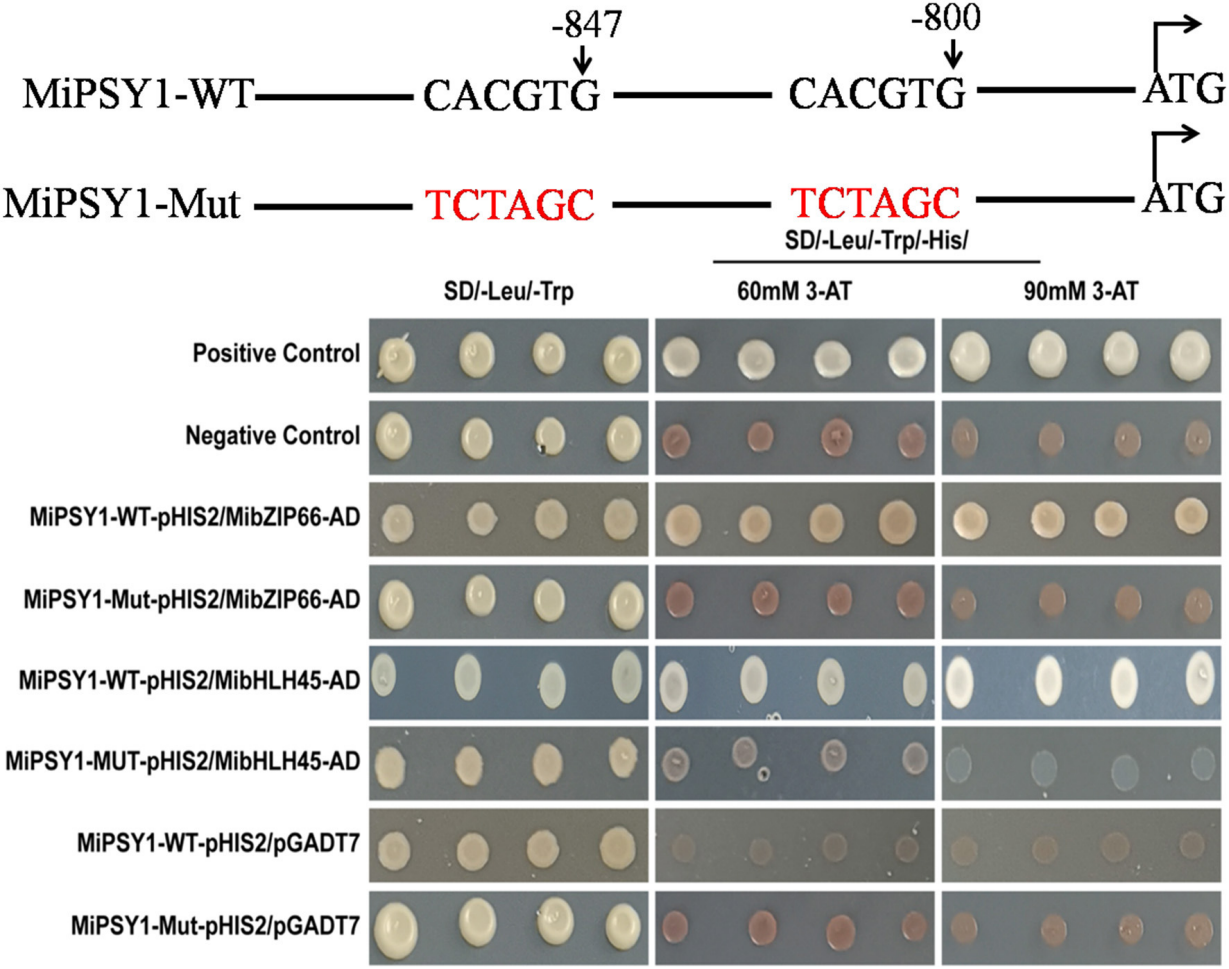
Yeast one-hybrid assay of MibZIP66 and MibHLH45 binding to the actual (WT) and mutated (Mut) promoters of MiPSY1.

The PlantTFDB analysis suggested that MibZIP66 and MibHLH45 could bind to the CACGTG sequence in the *MiPSY1* promoter, which was consistent with previous studies reporting that many bZIP and bHLH TFs specifically bind to the ACGT-containing elements, such as G-boxes (Pires and Dolan, 2010; Wolfgang et al., 2018). In our study, two CACGTG sequences were found to be located between the positions 852 and 800 bp, upstream of the transcriptional start codon in *MiPSY1* (Supplementary Table 13). YIH experiments and *cis*-element mutagenesis were performed. As shown in Figure 8, all of the yeast cells could grow normally on two dropout minimal media (SD/-Leu/-Trp). Nevertheless, only the yeast cells co-transformed with the positive control, pGADT7-MibZIP66+MibPSY1-WT promoter, or pGADT7-MibHLH45+MibPSY1-WT promoter grew well on selective medium SD/-His/Leu/-Trp supplemented with 60 or 90 mM 3-amino-1,2,4-triazole. The pGADT7-MibZIP66+MibPSY1-Mut promoter and pGADT7-MibHLH45+MibPSY1-Mut promoter could not grow on the selective medium when the two G-box elements CACGTG of the *MiPSY1* promoter were both mutated to TCTAGC (Figure 8). These results suggest that MibZIP66 and MibHLH45 could specifically bind to the G-box motif (CACGTG) of the *MiPSY1* promoter to regulate the expression of *MiPSY1* in the mango flesh.

## Discussion

Ever since the *Vitis vinifera* genome sequencing was completed in 2007 (Jaillon et al., 2007), the whole genomes of many fruit crops, including fruit trees have been fully sequenced. However, cultivars have experienced frequent recombination and exchange of genetic fragments, and the contraction and expansion of gene families during domestication and genetic improvement through breeding and intense artificial selection. Hence, a single genome may not fully display the genetic diversity of the species. Recently, genome assemblies for the two mango cultivars “Hong Xiang Ya” (Li et al., 2020) and “Alphonso” (Wang et al., 2020) have been published, providing insights on mango genome evolution. In the current study, the whole genome of the mango cultivar “Irwin” was sequenced using the third-generation PacBio technique, which generated a high-quality genome with contig N50 and 1.03 Mb long. The “Irwin” genome provides a valuable new resource to broaden the currently available mango genome data, permitting us to deepen our knowledge on comparative genomics of mango.

Mango fruit flesh comes in different colors, including milk-white, yellow, and deep yellow. Our results showed that the β-carotene content was significantly higher in “Irwin” flesh than in “Baixiangya” flesh during the fruit ripening process, suggesting that the accumulation levels of β-carotene were related to the deepness of flesh yellow color (Vásquez-Caicedo et al., 2005). The transcriptome analysis revealed that upstream genes of the β-carotene biosynthesis pathway, including *MiDXS, MiPSY*, and carotenoid decomposition genes (*MiCCD*) were expressed differentially between “Irwin” and “Baixiangya” flesh, leading us to speculate that their expression levels were contributed to the differential flesh β-carotene concentration between the two cultivars. *PSY* activity is generally accepted as the first committed step of carotenoid biosynthesis in fruit such as loquat (Fu et al., 2012) and apricot (Marty et al., 2005). We found that *MiPSY1* was expressed at significantly higher levels in “Irwin” than in “Baixiangya” during fruit development and ripening and that it was positively related to β-carotene production, suggesting the importance of its role. *LCYB* has been reported to be the rate-limiting enzyme for β-carotene biosyntheses, such as in durian fruits (Wisutiamonkul et al., 2017) and citrus fruits (Lu et al., 2018). However, in the present study, *MiLCYB* exhibited expression patterns uncorrelated with β-carotene accumulation during fruit development and ripening. Thus, we infer that *MiPSY1* acts as a key control point for β-carotene biosynthesis in the mango fruit flesh.

Transcription factors play a critical role in regulating functional gene expression to control the development of important traits in plants. A few TFs have been reported to directly regulate *PSY* expressions, such as RIN in tomato (Martel et al., 2011) and PIF1 in *Arabidopsis* (Toledo-Ortiz et al., 2010). In the present study, we found that the expression of *MibZIP66* and *MibHLH45* correlated with both *MiPSY1* expression and β-carotene content, suggesting that the two TFs might be associated with β-carotene regulation. bHLH TFs are known to regulate carotenoid biosynthesis. In papaya, CpbHLH1/2 transactivates the expression of *CpCYC-B* and *CpLCY-B* during fruit ripening (Zhou et al., 2019). Moreover, bZIP family TFs have been shown to be involved in the regulation of anthocyanin biosyntheses, such as *Arabidopsis* HY5 (Nawkar et al., 2017), tomato SIHY5 (Liu et al., 2018), and pear PybZIPa (Liu et al., 2019). However, bZIP TFs have rarely been proven to directly regulate the structural genes involved in carotenoid production. Our results verified that MibZIP66 and MibHLH45 could specifically bind and transactivate the *MiPSY1* promoter. A recent study has identified that TF–TF interactions can participate in carotenoid biosyntheses, such as CpEIN3a interacting with CpNAC2 in papaya (Fu et al., 2017), and SlNAC4 interacts with RIN in tomato (Zhu et al., 2014). However, the dual-luciferase assay showed that there was no significant synergistic effect of MibZIP66 and MibHLH45 on the *MiPSY1* promoter. Thus, MibZIP66 and MibHLH45 regulate the expression of *MiPSY1* to vary the content of β-carotene, thereby affecting the color formation in the mango flesh.

## Conclusions

In summary, the “Irwin” genome and the transcriptional regulation of *MiPSY1* during mango fruit ripening are valuable to expand our understanding of the genetic basis of flesh color development. These genomic resources are expected to help accelerate the genetic improvement of mangoes.

## Data Availability Statement

The raw sequences data reported in this paper have been deposited in the Genome Sequence Archive (GSA) in national genomics data center (https://ngdc.cncb.ac.cn), under accession number CRA004336.

## Author Contributions

XM, XL, YW, and JZ designed the study and performed the genome assembly and genome annotation. TB and JS edited the manuscript. BZ, WX, and LL collected the samples. SW and HW provided advice on the experimental design. All authors contributed to the article and approved the submitted version.

## Funding

This work was supported by the National Science Foundation of China (Grant No. 31672103), Guangdong Provincial National Science Foundation (No. 2021A1515010966), and Guangdong Provincial Special Fund for Modern Agriculture Industry Technology Innovation Teams (2019KJ108). We thank Dr. Mingjun Li (College of Horticulture, Northwest A & F University, Shanxi, China) for English editing.

## Conflict of Interest

The authors declare that the research was conducted in the absence of any commercial or financial relationships that could be construed as a potential conflict of interest.

## Publisher's Note

All claims expressed in this article are solely those of the authors and do not necessarily represent those of their affiliated organizations, or those of the publisher, the editors and the reviewers. Any product that may be evaluated in this article, or claim that may be made by its manufacturer, is not guaranteed or endorsed by the publisher.
