# Supplementary material for: Chromosome-Scale Genome and Comparative Transcriptomic Analysis Reveal Transcriptional Regulators of β-Carotene Biosynthesis in Mango
Source: Front Plant Sci. 2021 Oct 12;12:749108. doi: 10.3389/fpls.2021.749108 (PMC8545804; doi:10.3389/fpls.2021.749108)
Supplement: Supplementary file 1 [file Data_Sheet_1.ZIP › Supplementary file/Supplementary file 1-TableS1-S13.docx]

**Table S1.** Statistics of mango genome sequencing.

| Library | Data (Gb) | Depth  (×) | Q20 (%) | Q30 (%) |
| --- | --- | --- | --- | --- |
| Mango | 20.26 | 61.96 | 96.81 | 93.26 |

**Table S2.** Statistics of 17-mer analysis.

| Sample | Kmer | Peak  depth | Genome size (Mbp) | Used bases | Used read  number |
| --- | --- | --- | --- | --- | --- |
| Mango | 17 | 52 | 327 | 18,108,083,950 | 17,000,539,292 |

**Table S3.** Hi-C library details.

| Species | Base Number | Average  depth | %≥Q  30 | Total Read  Pairs | Mapped reads (%) | Unique Mapped reads (%) |
| --- | --- | --- | --- | --- | --- | --- |
| Mango | 41,707,497,172 | ~× | 92.14 | 139,496,309 | 243,464,536  (87.27) | 89,028,247  (63.82) |

**Table S4.** Summary of mango pseudomolecules.

| Names | Cluster Number | Cluster Length (bp) | Order Number | Order Length (bp) |
| --- | --- | --- | --- | --- |
| LG1 | 38 | 20605751 | 25 | 19858944 |
| LG2 | 88 | 29814655 | 51 | 27904229 |
| LG3 | 124 | 27421305 | 39 | 21547171 |
| LG4 | 57 | 23585481 | 35 | 22219900 |
| LG5 | 104 | 23793343 | 44 | 19366160 |
| LG6 | 58 | 19702075 | 34 | 18084944 |
| LG7 | 44 | 18896851 | 29 | 18085770 |
| LG8 | 49 | 19104996 | 27 | 17851717 |
| LG9 | 40 | 18751278 | 23 | 17213665 |
| LG10 | 29 | 17291138 | 16 | 16657565 |
| LG11 | 93 | 16803741 | 35 | 12873895 |
| LG12 | 32 | 16515692 | 18 | 15651807 |
| LG13 | 11 | 15185531 | 8 | 15017632 |
| LG14 | 16 | 15124146 | 11 | 14873568 |
| LG15 | 38 | 14744871 | 22 | 13848496 |
| LG16 | 40 | 14744871 | 22 | 13702534 |
| LG17 | 34 | 14961586 | 15 | 13424108 |
| LG18 | 30 | 14379947 | 20 | 12881312 |
| LG19 | 23 | 13458673 | 15 | 12203376 |
| LG20 | 54 | 12755550 | 31 | 20959595 |
| Total (Ratio %) | 1002 (97.19) | 375276096 (99.51) | 520(51.9) | 344226388(91.73) |

**Table S5.** The mapping rate of RNA-seq data.

| Names | Totals reads | Mapped reads | Mapping rate (%) |
| --- | --- | --- | --- |
| BSY1-1 | 56,086,668 | 51,870,244 | 92.48 |
| BSY1-2 | 45,714,608 | 42,345,159 | 92.63 |
| BSY1-3 | 41,492,078 | 38,381,475 | 92.50 |
| BSY2-1 | 45,638,552 | 42,554,660 | 93.24 |
| BSY2-2 | 40,846,258 | 37,897,307 | 92.78 |
| BSY2-3 | 42,367,638 | 39,378,879 | 88.51 |
| BSY3-1 | 53,644,764 | 50,650,699 | 94.42 |
| BSY3-2 | 42,662,562 | 40,415,559 | 94.73 |
| BSY3-3 | 52,671,586 | 49,977,821 | 94.89 |
| IRS1-1 | 44,513,514 | 42,507,413 | 95.49 |
| IRS1-2 | 41,768,468 | 38,983,286 | 93.33 |
| IRS1-3 | 50,735,072 | 48,427,383 | 95.45 |
| IRS2-1 | 38,510,972 | 34,771,914 | 90.29 |
| IRS2-2 | 45,727,746 | 43,313,068 | 94.72 |
| IRS2-3 | 43,832,220 | 39,804,760 | 90.81 |
| IRS3-1 | 45,529,566 | 43,643,023 | 95.86 |
| IRS3-2 | 47,524,818 | 45,534,929 | 95.81 |
| IRS3-3 | 47,157,642 | 45,208,828 | 95.87 |

**Table S6.** BUSCO analysis of genome assembly.

| Description | Number  (eudicotyledons) | Percentage(%) |
| --- | --- | --- |
| Complete BUSCOs (C) | 1991 | 93.8 |
| Complete and single-copy BUSCOs (S) | 1651 | 77.8 |
| Complete and duplicated BUSCOs (D) | 340 | 16.0 |
| Fragmented BUSCOs (F) | 83 | 3.9 |
| Missing BUSCOs (M) | 47 | 2.3 |
| Total BUSCO groups searched | 2121 | 100 |

**Table S7.** Detail Statistics of repetitive elements.

|  | Count | Length (bp) | % of genome |
| --- | --- | --- | --- |
| DNA | 43946 | 23588076 | 6.25455 |
| LINE | 1617 | 2141074 | 0.567721 |
| SINE | 85 | 6969 | 0.00184788 |
| LTR | 57142 | 58408329 | 15.4874 |
| Other | 207051 | 12785412 | 3.39014 |
| Unknown | 152898 | 48150323 | 12.7674 |
| Total | 462739 | 138210280 | 36.64 |

**Table S8.** Gene model statistics.

| Description | mean |
| --- | --- |
| Gene_length | 3409 |
| cDNA_length | 1570 |
| exon_number | 5.5 |
| CDS_length | 1142 |
| CDS_num | 5 |
| Intron_length | 1825 |

**Table S9.** Expression patterns of candidate transcription factors in MElightcyan module.

| Gene name | TF type | BYS1 | BYS2 | BYS3 | IRS1 | IRS2 | IRS3 |
| --- | --- | --- | --- | --- | --- | --- | --- |
| Mango_gene00779 | C2C2-LSD | 22.10 | 39.43 | 35.12 | 28.64 | 63.55 | 105.06 |
| Mango_gene01565 | NAC | 14.28 | 76.72 | 237.50 | 37.54 | 13.25 | 317.16 |
| Mango_gene01895 | WRKY | 4.46 | 30.39 | 38.63 | 39.76 | 3.21 | 283.54 |
| Mango_gene01983 | C2C2-CO-like | 1.70 | 2.41 | 0.19 | 1.90 | 3.08 | 10.14 |
| Mango_gene02050 | AP2/ERF-ERF | 17.35 | 0.29 | 0.32 | 9.65 | 4.00 | 55.21 |
| Mango_gene02270 | FAR1 | 2.28 | 1.86 | 0.70 | 2.40 | 2.59 | 0.46 |
| Mango_gene02341 | Trihelix | 1.33 | 3.32 | 0.72 | 1.64 | 5.37 | 10.71 |
| Mango_gene04108 | MYB-related | 9.22 | 15.80 | 4.62 | 12.80 | 11.23 | 3.31 |
| Mango_gene04754 | AP2/ERF | 5.54 | 4.73 | 0.56 | 4.96 | 4.21 | 0.08 |
| Mango_gene04947 | FAR1 | 7.26 | 7.77 | 1.27 | 9.27 | 8.40 | 1.98 |
| Mango_gene06125 | NAC | 0.27 | 0.12 | 0.09 | 1.45 | 0.04 | 4.63 |
| Mango_gene06587 | C2C2-Dof | 0.29 | 0.25 | 14.41 | 0.57 | 7.00 | 259.70 |
| Mango_gene08233 | LOB | 9.54 | 39.89 | 54.98 | 55.66 | 188.51 | 295.73 |
| Mango_gene08925 | Tify | 13.57 | 9.64 | 31.31 | 15.41 | 14.07 | 47.08 |
| Mango_gene09426 | MYB | 1.01 | 1.20 | 23.46 | 1.48 | 8.71 | 70.42 |
| Mango_gene11643 | GARP-G2-like | 0.00 | 0.02 | 6.02 | 0.04 | 0.40 | 4.10 |
| Mango_gene12977 | C2H2 | 3.76 | 14.45 | 6.71 | 24.74 | 9.03 | 27.29 |
| Mango_gene13239 | FAR1 | 1.57 | 4.18 | 4.72 | 3.60 | 3.91 | 12.60 |
| Mango_gene13350 | NAC | 33.36 | 21.37 | 88.86 | 18.26 | 8.72 | 120.73 |
| Mango_gene16549 | AP2/ERF | 4.36 | 1.02 | 0.81 | 0.11 | 3.75 | 8.27 |
| Mango_gene17414 | bHLH | 8.56 | 10.84 | 0.02 | 7.71 | 7.71 | 0.06 |
| Mango_gene17464 | bHLH | 2.75 | 0.04 | 4.27 | 5.33 | 0.32 | 10.76 |
| Mango_gene17764 | NAC | 11.34 | 6.43 | 20.09 | 41.47 | 15.33 | 115.52 |
| Mango_gene19166 | C2H2 | 1.21 | 0.31 | 0.00 | 0.41 | 1.79 | 12.31 |
| Gene name | TF type | BYS1 | BYS2 | BYS3 | IRS1 | IRS2 | IRS3 |
| Mango_gene20114 | LOB | 0.05 | 0.00 | 2.61 | 0.05 | 0.00 | 9.74 |
| Mango_gene20245 | MYB-related | 12.46 | 13.46 | 22.27 | 13.47 | 6.31 | 51.52 |
| Mango_gene20675 | bHLH | 4.13 | 2.89 | 8.06 | 5.73 | 4.93 | 88.03 |
| Mango_gene22327 | PLATZ | 0.42 | 27.71 | 63.69 | 11.40 | 13.03 | 99.19 |
| Mango_gene23131 | Tify | 0.24 | 0.15 | 1.32 | 1.80 | 0.15 | 3.28 |
| Mango_gene24734 | MYB | 0.07 | 0.02 | 0.04 | 0.75 | 0.07 | 7.25 |
| Mango_gene25775 | TCP | 6.24 | 6.43 | 24.94 | 3.26 | 6.54 | 32.43 |
| Mango_gene26992 | C2H2 | 10.62 | 1.60 | 0.25 | 1.77 | 12.48 | 31.27 |
| Mango_gene27089 | bHLH | 5.71 | 6.67 | 0.15 | 5.76 | 3.85 | 0.47 |
| Mango_gene27181 | WRKY | 33.03 | 16.29 | 38.55 | 6.01 | 4.19 | 68.68 |
| Mango_gene29098 | bHLH | 6.26 | 8.55 | 0.02 | 9.52 | 6.15 | 0.01 |
| Mango_gene29238 | Tify | 0.59 | 0.08 | 0.38 | 0.54 | 0.97 | 19.96 |
| Mango_gene29253 | NAC | 18.20 | 7.44 | 28.20 | 52.06 | 19.59 | 149.47 |
| Mango_gene30145 | bHLH | 0.35 | 0.48 | 8.02 | 1.29 | 0.29 | 23.61 |
| Mango_gene30775 | MYB | 1.01 | 0.00 | 0.15 | 0.02 | 0.32 | 39.83 |
| Mango_gene30794 | bHLH | 2.03 | 2.44 | 6.08 | 3.26 | 5.18 | 59.01 |
| Mango_gene31064 | bHLH | 17.36 | 20.61 | 0.14 | 19.85 | 14.61 | 0.97 |
| Mango_gene32384 | NF-YB | 37.51 | 28.14 | 14.41 | 22.29 | 21.68 | 88.45 |
| Mango_gene32772 | HSF | 2.48 | 4.26 | 7.16 | 23.11 | 7.28 | 73.02 |
| Mango_gene34777 | AP2/ERF | 0.05 | 0.24 | 6.25 | 0.17 | 14.96 | 32.60 |
| Mango_gene35272 | PLATZ | 34.71 | 40.53 | 27.16 | 20.03 | 27.98 | 425.80 |
| Mango_gene35447 | bZIP | 11.76 | 12.17 | 4.33 | 9.40 | 12.73 | 1.54 |
| Mango_gene36365 | WRKY | 24.63 | 29.23 | 0.04 | 23.10 | 18.00 | 0.60 |
| Mango_gene36543 | HB-KNOX | 15.40 | 17.24 | 0.01 | 14.20 | 10.48 | 0.36 |

**Table S10.** Primers used in this study.

| Genes  (full length or fragments) | Forward Primer (5’-3’) | Reverse Primer (5’- 3’) | Used for |
| --- | --- | --- | --- |
| MiPSY1 | ATGTCTGTGGCTTTGCTATGG | TCAATTTTGCACTTTTAACAGTGC | CDS cloning |
| MiPSY1 | GGATCCATGTCTGTGGCTTTGCTATGG | GAATTCTCAATTTTGCACTTTTAACAGTG | Promoter cloning |
| pGreenII 62-SK- MibZIP66 | GGCCGCTCTAGAACTAGTGGATCC  ATGGGGATTCAGACAATGGGGTC | ATCGATAAGCTTGATATCGAATTC  TTAGAATGAGGCTGATGTCGTTC | Dual luciferase assay |
| pGreenII 62-SK- MibZIP36 | GGCCGCTCTAGAACTAGTGGATCCA  TGGGGAGTAACATAAACTTCAAG | ATTTCAGCGTACCGAATTGGTAC  CTCACCATGGACCAGTCTGTGT |  |
| pGreenII 62-SK- MibHLH105 | GGCCGCTCTAGAACTAGTGGATC  CATGGCTTCACCTGATAATCCA | ATTTCAGCGTACCGAATTGGTA  CCTTAAGCAACTGGTGGGCG |  |
| pGreenII 62-SK- MibHLH31 | GGCCGCTCTAGAACTAGTGGAT  CCATGGATCCGCCAGCAAT | ATTTCAGCGTACCGAATTGGTACCT  TAAGTCATTCTTTCAAAACCACC |  |
| pGreenII 62-SK- MibHLH64 | GGCCGCTCTAGAACTAGTGGAT  CCATGAATAGAGGAGTGGTGCC | ATTTCAGCGTACCGAATTGGTACC  TCACATCTCCATCTTTAGATTGC |  |
| pGreenII 62-SK- MibHLH45 | GGCCGCTCTAGAACTAGTGGATC  CATGTCTCACATAGCTGTGGAGAGA | ATTTCAGCGTACCGAATTGGTACCT  TACATCTCGTGGGAGTAAACAAC |  |
| pGreenII 62-SK- MibHLH130 | GGCCGCTCTAGAACTAGTGGATCCAT  GGATTCAAGTAGTAGTCATAATTTTCA | ATTTCAGCGTACCGAATTGGTACC  CTAACCCCTATTCATCCTTTTCCT |  |
| pGreenII 0800-LUC-MiPSY1 pro | GGCCGCTCTAGAACTAGTGGAT  CCATGTCTGTGGCTTTGCTATGG | ATCGATAAGCTTGATATCGAATTC  TCAATTTTGCACTTTTAACAGTG |  |
| pHIS2- MiPSY1pro | ACGACTCACTATAGGGCGAATT  CAAATAAAGTTTTCTTCCTGC | CGCGGATCGATTCGCGAACGCG  TATATAATTAATTTGACCTTTA | Y1H |
| pHIS2- MiPSY1pro-Mut | ACGACTCACTATAGGGCGAATT  CAAATAAAGTTTTCTTCCTGC | CGCGGATCGATTCGCGAACGCG  TATATAATTAATTTGACCTTTA |  |
| pGADT7- MibZIP66 | CATATGGCCATGGAGGCCAGTGAAT  TCATGGGGATTCAGACAATGGGGTC | CATCTGCAGCTCGAGCTCGATGGATC  CTTAGAATGAGGCTGATGTCGTTC |  |
| pGADT7- MibHLH45 | CATATGGCCATGGAGGCCAGTGAA  TTCATGTCTCACATAGCTGTGGAG | CATCTGCAGCTCGAGCTCGATGGA  TCCTTACATCTCGTGGGAGTAAAC | YIH |
| MiPSY1 | TTTTGCCTGGAACTTTGAGC | TTCATCCGTCCTCCTACACC | Real-time PCR |
| MibZIP66 | GACTTTGGAGGATTTCTTGGTG | CATGTGCAGTGGCTGTGGTAT |  |
| MibZIP36 | CGGCAACCGTCGATCTATTC | TTACCACCTCCGCCACTTCC |  |
| MibHLH105 | GTGATGAGAAACAAAGGCTAAAGA | CCACATAGAAACTCCAGGACAAC |  |
| MibHLH31 | CCACCAGAGCCACCTAAACA | TTGGAGGAAACACCTCAATACC |  |
| MibHLH64 | GTTTGTTGGGTGGTGGTGAC | TCCTTGAAATGGCATAACTGG |  |
| MibHLH45 | TCTCGGGATCAAACGTCATA | GTGGGAGTAAACAACATCTGGAAA |  |
| MibHLH130 | TGCTGGATTCCCGTATGGTT | GGCAATGCTTCGAGGGTGAG |  |
| Actin | TTGTGCTGGATTCTGGTG | AGCAGTGGTGGTGAACAT |  |
| MiPSY1 | AGTGGATCCCCCGGGCTGCAG  ATGTCTGTGGCTTTGCTA | GGGCCCCCCCTCGAGGTCGAC  ATTTTGCACTTTTAACAG | Subcellular localization |
| MibZIP66 | AGTGGATCCCCCGGGCTGCAG ATGGGGATTCAGACAATG | GGGCCCCCCCTCGAGGTCGAC GAATGAGGCTGATGTCGTTCT |  |
| MibHLH45 | AGTGGATCCCCCGGGCTGCAG ATGTCTCACATAGCTGTG | GGGCCCCCCCTCGAGGTCGAC CATCTCGTGGGAGTAAACAACA |  |

**Table S11.** Sequences (5′–3′) for *MiPSY1* CDS of ‘Irwin’.

ATGTCTGTGGCTTTGCTATGGGTTGTATCTCCCAACTCACAGTTGTCAAGATATGCTGGATTTGATGATTCAGTTCGTGATGGAAACCGACTAATAGACTCATCAAGATTTCTGTACCGGGATCGTGGTTCGATGTTTAGCTGTAGACCTAATAAGGATAGGAAACAGAAATGGAATTCTTGCTCATTTGGTACAGATTTTAGGCATCCTTGCTTAGGTGGAATCAACTTACCTGAGATATCATGTATGGTGGCTAGCACAGTGGGAGAAATGGCCGTGTCTTCAGAAGAAAAGGTTTACAATGTGGTGTTGAAGCAGGCAGCCTTGGTTAATAGGCAATTAAGGTCAAGTGGAGAACTTGATGTGAAACCTGATCTTGTTTTGCCTGGAACTTTGAGCTTGTTGAGTGAAGCTTATGATCGGTGTGGAGAAGTTTGTGCAGAGTATGCCAAGACATTTTACTTGGGAACTCTCCTAATGACTCCTGAAAGAAGAAGAGCTATATGGGCTATATATGTGTGGTGTAGGAGGACGGATGAACTTGTTGATGGACCTAATGCTTCACACATTACTCCAACAGCCTTAGATAGATGGGAATCCAGGTTGGAAGATGTTTTCCAGGGTCGTCCATTTGACATGCTTGATGCTGCTTTATCAGATACAGTAGCAAAATTTCCTGTTGACATTCAGCCATTTAGAGACATGATTGAAGGAATGAGGATGGACCTTAAGAAATCAAGATATAAGAACTTTGATGAACTATACCTGTATTGTTATTATGTTGCTGGCACTGTTGGTTTAATGAGTGTTCCAGTCATGGGAATTGCACCTGGATCACAGGCAACAACAGAGAGTGTCTACAATGCTGCCTTGGCATTAGGGATTGCTAACCAGCTCACCAACATACTCCGGGATGTTGGAGAGGATGCAAGAAGAGGAAGGGTTTATCTACCACAAGATGAGCTGGCACAAGCAGGGCTTTCTGATGAAGACATATTTGCTGGAAAGGTGTCTGCTAAATGGAGAAATTTCATGAAGAACCAAATTGAGAGAGCAAGGATGTTCTTTGATGAAGCAGAGAAAGGCGTGGCAGAGCTGAGCGATGCTAGCAGATGGCCGGTATGGGCATCGTTGCTTTTGTATCGTCAAATACTAGACGAGATTGAAGCAAATGATTATGACAATTTCACAAGGAGGGCATATGTGAGTAAAGTGAAGAAGATAGCTGCATTGCCAGTCTCCTATACAAGGTCGCTCGTACGCCCCTCCAGAAAAGCACTGTTAAAAGTGCAAAATTGA

**Table S12.** Sequences (5′–3′) for *MiPSY1* CDS of ‘Baixiangya’.

ATGTCTGTGGCTTTGCTATGGGTTGTATCTCCCAACTCACAGTTGTCAAGATATGCTGGATTTGATGATTCAGTTCGTGATGGAAACCGCCTAATAGACTCATCAAGATTTCTGTACCGGGATCGTGGTTCGATGTTTAGCTGTAGACCTAATAAGGATAGGAAACAGAAATGGAATTCTTGCTCATTTGGTACAGATTTTAGGCATCCTTGCTTAGGTGGAATCAACTTACCTGAGATATCATGTATGGTGGCTAGCACAGTGGGAGAAATGGCCGTGTCTTCAGAAGAAAAGGTTTACAATGTGGTGTTGAAGCAGGCAGCCTTGGTTAATAGGCAATTAAGGTCAAGTGGAGAACTTGATGTGAAACCTGATCTTGTTTTGCCTGGAACTTTGAGCTTGTTGAGTGAAGCTTATGATCGGTGTGGAGAAGTTTGTGCAGAGTATGCCAAGACATTTTACTTGGGAACTCTCCTAATGACTCCTGAAAGAAGAAGAGCTATATGGGCTATATATGTGTGGTGTAGGAGGACGGATGAACTTGTTGATGGACCTAATGCTTCACACATTACTCCAACAGCCTTAGATAGATGGGAATCCAGGTTGGAAGATGTTTTCCAGGGTCGTCCATTTGACATGCTTGATGCTGCTTTATCAGATACAGTAGCAAAATTTCCTGTTGACATTCAGCCATTTAGAGACATGATTGAAGGAATGAGGATGGACCTTAAGAAATCAAGATATAAGAACTTTGATGAACTATACCTGTATTGTTATTATGTTGCTGGCACTGTTGGTTTAATGAGTGTTCCAGTCATGGGAATTGCACCTGGATCACAGGCAACGACAGAGAGTGTCTACAATGCTGCCTTGGCATTAGGGATTGCTAACCAGCTCACCAACATACTCCGGGATGTTGGAGAGGATGCAAGAAGAGGAAGGGTTTATCTACCACAAGATGAGCTGGCACAAGCAGGGCTTTCTGATGAAGACATATTTGCTGGAAAGGTGTCTGCTAAATGGAGAAACTTCATGAAGAACCAAATTGAGAGAGCAAGGATGTTCTTTGATGAAGCAGAGAAAGGCGTGGCAGAGCTGAGCGATGCTAGCAGATGGCCGGTATGGGCATCGTTGCTTTTGTATCGTCAAATACTAGACGAGATTGAAGCAAATGATTATGACAATTTCACAAGGGGGGCATATGTGAGTAAAGTGAAGAAGATAGCTGCATTGCCAGTCTCCTATGCAAGGTCGCTCGTACGCCCCTCCAGAAAAGCACTGTTAAAAGTGCAAAATTGA

**Table S13.** Sequences (5′–3′) for MiPSY1 promoter of ‘Irwin’.

AAATAAAGTTTTCTTCCTGCTCAAAACCGAGACAAATTTAGACTCTTTCTCCTACAAGTTAAAACCACAACAGCACTTACTATTTTCAATATTATTTTATTATAATTAACTCACCTCCATTACCTTCTTGCCTTCCTCCATCCCATGAAAGTTGGCATTTATCAATACGTGTCAAACACTTGACCATGTGAACCCATGGACGACAGGTCGTCTATTCATATTTTATTTCTGTATTTGAAATAAAAGACAAATGCCTTTCTCCATTTCCACTTTTTCACTCTTCTGGGCTCCACCACATCCTCCCGGGATCTAGCTTTTCTTTTCCCTCCTCACTCAGCGCTTGCCACGTGAACTCTCACCTCCACTCTTCCTCCCGATAACCTTATCGTCCACGTGGGATCCTCTTCTCTCTTCTACTTTTCACTCGGGTCGGGTAAATTTATTAACACTTTATATGAAAAAGATTGAATTTGATGTTTTTATTTTCTTAAAATATTTGACCAGTCAACGATGATAAAGACTTCCTTAATTAATACATTTTAGAATATTAAATATTGAAAACAAAATGCGAAAAATAGAGAATAATCCTTTTAAAAAAGGCATATTAATTAAGTAAATTTTTCTTAATTAATTTTGTAACTGAAATATAGATATATTTTTTTAAAAACAATTTCACCCATATCGGATCACTTTTTAAAGGTCAAATTAATTATATTAAGTAAACCAAATATCAGATTTAGTGATTGATTTGAAAGTCACTTAATCAAATAAATCAAAGTTTGATTTTAATATATTATTAAAAAAAATTTAATTCATATTTTCTTTTATTTATAGTCTTCTCCACATATTTTTTATATTAATTTCTTTTTTGTTAAAATAAAACTTTTATTCTTATTTCACAAATACAAAACCCAATAAACCAACATATTTTTTTTGAAGTGATTTAAAAAAATACTCTTTAAAAATAAAAAAATTAAATTGGTTTAATAAATATTTAATTTTCAACCTTTTGGAAATTTTAGAACCGCCTATATTGTTAATTAGGTTATTGTTATTTTTTTAAATTCAAATATGGAGAAGGAAACGGGTTATTCAAGAATATCCATATTCCATCCGGATCTGAAATCTGATACATGACAAAACAGTAACCATATGGATAAAAAAGAGGAAAGAGAGGCCACTCTGCAAATAAATTC**ATG**
