# Supplementary material for: Chromosome-Scale Genome and Comparative Transcriptomic Analysis Reveal Transcriptional Regulators of β-Carotene Biosynthesis in Mango
Source: Front Plant Sci. 2021 Oct 12;12:749108. doi: 10.3389/fpls.2021.749108 (PMC8545804; doi:10.3389/fpls.2021.749108)
Supplement: Supplementary file 1 [file Data_Sheet_1.ZIP › Supplementary file/Supplementary file 2-FigureS1-S3.docx]

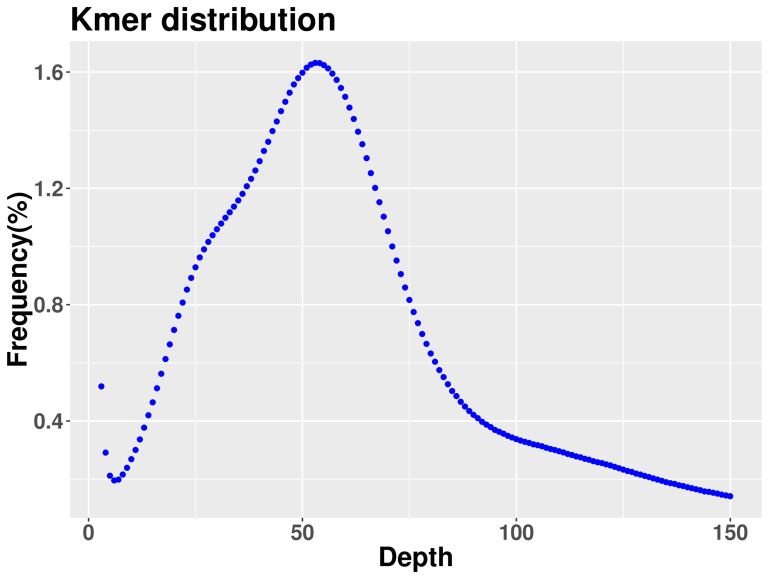


**Fig. S1** Distribution of 17-mer frequency.

**
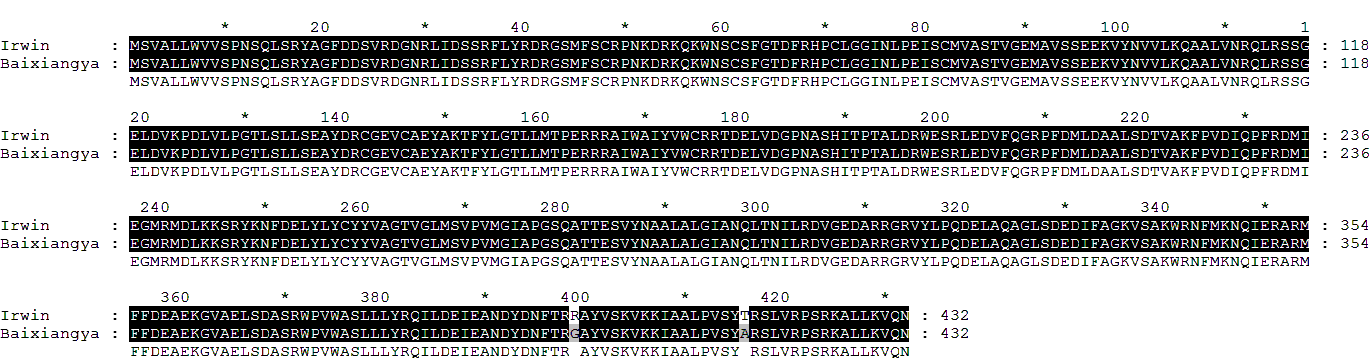
**

**Fig. S2** The deduced amino acid sequences for MiPSY1 (Mango_gene22766) of ‘Irwin’ and ‘Baixiangya’.

**(A)**


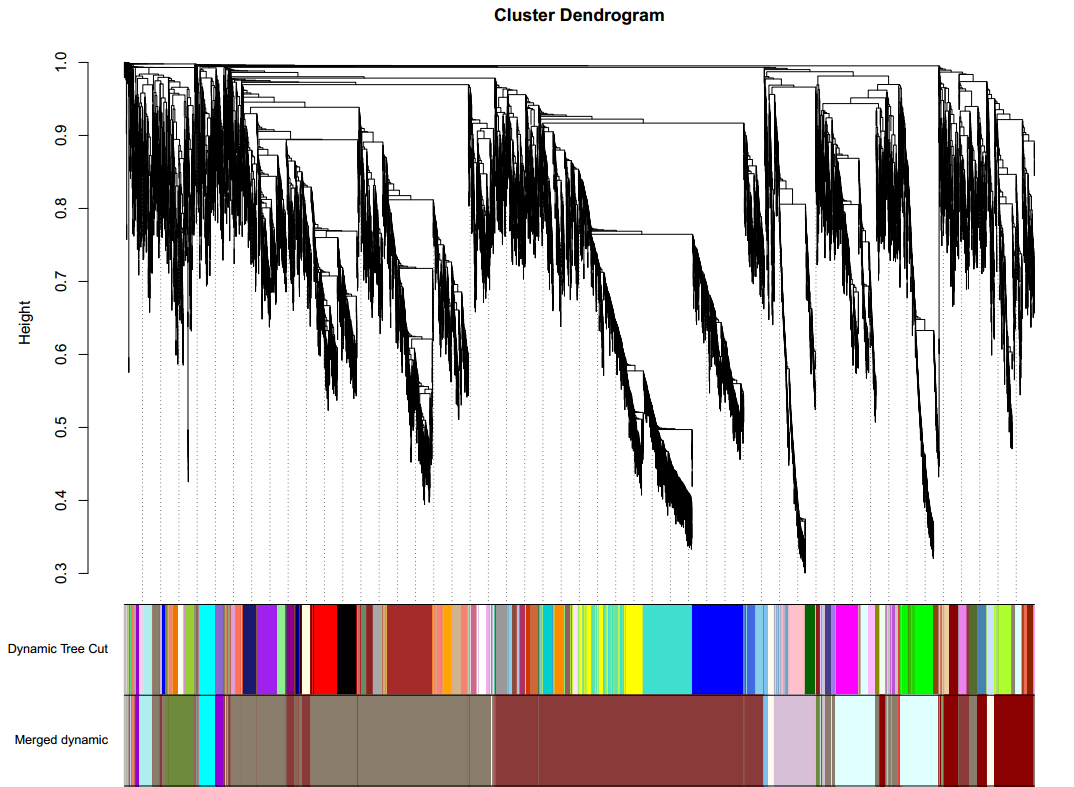


**(B)**


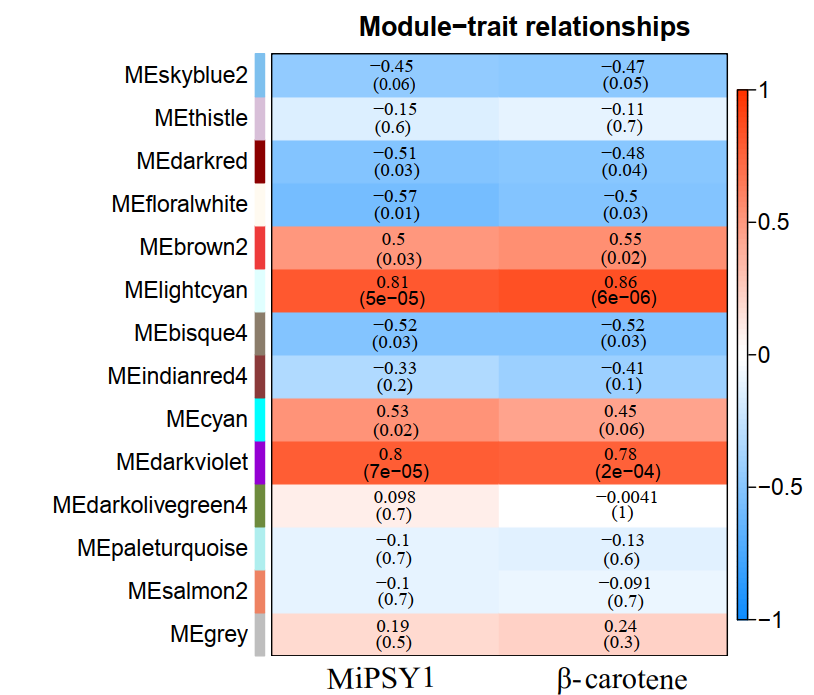


**Fig. S3** Weighted gene co-expression network analysis (WGCNA) of DEGs identified in mango flesh during fruit development and ripening. (A) Hierarchical cluster tree showing in14 modules by a major tree branch; (B) Module-trait correlations and corresponding *p*-values ( in parentheses).
